# buzzdetect: an open-source deep learning tool for automated bioacoustic pollinator monitoring

**DOI:** 10.1093/jisesa/ieaf104

**Published:** 2025-12-10

**Authors:** Luke E Hearon, Lillian H P Johnson, James Underwood, Chia-Hua Lin, Reed M Johnson

**Affiliations:** Department of Entomology, The Ohio State University, Columbus, OH, USA; Department of Entomology, The Ohio State University, Columbus, OH, USA; Department of Computer Science, Dartmouth College, Hanover, NH, USA; Department of Entomology, The Ohio State University, Columbus, OH, USA; Department of Entomology, The Ohio State University, Columbus, OH, USA

**Keywords:** machine-learning, AI, insect sampling, bee, *Apis mellifera*

## Abstract

Ecological studies of pollinators often require long-term and extensive monitoring, posing a significant cost and limitation to research. Traditional sampling methods of observation such as sweep netting and pan trapping provide valuable information on pollinator diversity, but scale poorly when conducting large sampling efforts across space and time. We introduce “buzzdetect,” a tool to apply deep learning models to audio data for passive acoustic monitoring of pollinators, and test our accompanying audio classification model. The model is capable of distinguishing the buzzing of insect flight from environmental noise on a second-by-second basis with a sensitivity of 28% and a precision of 95%. As a demonstration of the value of buzzdetect, we apply the tool to recordings from 4 crops and 1 wildflower. The results reveal differences in timing and intensity of foraging that correspond with prior literature: activity peaked earliest for chicory and latest for soybean, while total activity was higher in mustard and soybean than in the other crops.

## Introduction

Sampling effort is one of the largest costs of carrying out pollinator research. Ecological systems often have low signal-to-noise ratios, which demand extensive sampling to overcome. This issue is compounded for studies that seek to characterize patterns in space (e.g. edge effects, habitat fragmentation, population distribution) or in time (e.g. foraging patterns, population dynamics). Studying these effects requires sampling across many points in space or over longer periods of time. The labor and expertise required for sampling can quickly become the limiting factor of a study’s sample size, hampering the ability to discern patterns of pollinator activity with sufficient resolution.

Insect sampling methods can be generally divided into 2 categories: active and passive. Active methods require the researcher to be present during the course of sampling. Two of the most common active methods of pollinator monitoring are visual observation and sweep netting. Visual observation is appealing for the ability to confirm foraging events on flowers, ensuring that sampled insects are indeed pollinators of the plant in question and not incidental visitors. In contrast, sweep netting affords finer taxonomic resolution and has repeatedly been shown to capture a high abundance and richness of pollinators (Baum and Wallen 2011, Popic et al. 2013, Prendergast et al. 2020). The downside to both of these methods is their time-intensiveness. Because a researcher must be present for the entire duration of sampling, active methods are prohibitively costly for studies that are large in spatial or temporal scale. Passive methods capture insects without the attendance of the researcher. The most common passive method for pollinator monitoring is pan trapping, which allows a single researcher to deploy numerous bee bowls, leave them for the desired duration, and then retrieve and identify samples at leisure. This facilitates extensive sampling across points in space and can capture species across a time period of interest, but it does not improve temporal resolution. To distinguish patterns across time, the researcher must reset the traps at each time point. For example, to measure foraging on an hour-by-hour basis, the researcher must return to every study site at every hour to reset traps, which diminishes the advantage of passive monitoring and may be infeasible for large-scale studies. Further, the capture of pan traps is itself a function of local floral abundance. When there are few flowers in the local environment, the bowls, which imitate the color of flowers, become relatively more attractive than when surrounding resources are plentiful (Wilson et al. 2008, Baum and Wallen 2011, Popic et al. 2013, Kuhlman et al. 2021, Westerberg et al. 2021). This is a consequential flaw if floral abundance varies across study sites or as the result of an intervention. This bias can be so strong as to completely invert the measured trend of pollinator abundance. St Clair et al. (2020) found that the number of honey bees captured by pan traps in soybean fields was low during soybean bloom, then increased dramatically when flowering ended. This pattern is implausible, as the authors note, and indeed the opposite trend has been found when sampling with methods not suffering this bias (Souza et al. 2023, Forrester et al. 2024). Finally, after the bouts of sampling have concluded, both sweep netting and pan trapping generate samples of insects that require taxonomic expertise and a significant investment of time to process and identify.

Bioacoustic monitoring offers a promising solution to the above shortcomings. This method detects and identifies organisms by the sounds they produce, allowing recording devices to be deployed at scale as easily as pan traps while offering fine temporal resolution. Because recorders should not attract pollinators, their sampling is not confounded with resource abundance in the local landscape. Most importantly for large-scale studies, the analysis of audio data can be automated through the use of machine learning by building models that identify the sound signatures of species of interest. This is the basis of passive acoustic monitoring, which seeks to answer ecological questions through automated monitoring (Ross et al. 2023). Acoustic monitoring has seen rapid adoption in avian systems (Xie et al. 2023), aquatic systems (Parsons et al. 2022), and whole-soundscape analysis (Nieto-Mora et al. 2023, Turlington et al. 2024). A recent landmark in the field was the release of the Cornell Lab of Ornithology’s powerful avian identification model “BirdNET” (Kahl et al. 2021), which has received hundreds of citations in the few years since its publication—in no small part because of the free release of their model weights and accessible analysis software (BirdNET Team 2025).

Entomological bioacoustics has seen little development relative to these systems. Kohlberg et al. (2024) recently reviewed publications using bioacoustics for insect detection and identification. The authors identified 49 papers employing automated bioacoustics to monitor insect populations, the earliest being a floppy disk-based system in 1978, but the majority (28 papers) being published since 2020. Gradišek et al. (2017), Ribeiro et al. (2021), and Ferreira et al. (2023) tested a number of different algorithms capable of classifying recordings of bumble bee (*Bombus* spp.) flight or sonication buzzes according to species. However, while these models are impressive, they are not suitable for passive acoustic monitoring because they are not designed to distinguish insect sounds from environmental noise. Rather, they are designed to be run on input audio known to contain a bumble bee buzz. Additionally, to our knowledge, only the models from Ferreira et al. (2023) are publicly available (Ferreira 2025). There also exist some studies that have created models suitable for passive monitoring. Folliot et al. (2022) produced a convolutional neural network and Kawakita and Ichikawa (2019) trained a support vector machine, with both of these models capable of distinguishing insect buzzes from environmental noise. However, to the best of our knowledge, these models were not made public. The only reproducible, publicly available method capable of passive acoustic monitoring of flying insects to our knowledge was produced by Heise et al. (2017). This algorithm was deployed in a field setting by Miller-Struttmann et al. (2017), showing detection results that correlate with visual observation and seed yield. However, the original publication (Heise et al. 2017) reports variable performance, with precision reaching single digits. Further, we cannot find any code to apply the algorithm to audio data, though it could be constructed from the description in Heise et al. (2017). Two companies produce devices for passive acoustic monitoring of pollinators: Spectrum by 3Bee and the Pollination Insight Platform by BeeHero. Alberti et al. (2023) conducted passive acoustic monitoring of pollinators using the Spectrum device by 3Bee, finding a compelling trend of detections across the course of the day. While these companies’ products appear to be effective for passive acoustic monitoring of pollinators, they are offered as paid commercial services and do not currently appear to be available as standalone devices or as software for immediate purchase.

Aside from the algorithm produced by Heise et al. (2017), we are aware of no model, tool, or method that is freely available and is capable of conducting passive acoustic monitoring of pollinators. The goal of this work is to (i) produce and publish a machine learning model capable of detecting insect buzzes for pollinator monitoring, (ii) develop an accompanying free and open source tool to apply the model to large audio datasets, (iii) test the method using audio from 5 sources of forage [pumpkin, *Cucurbita pepo* L.; chicory, *Chicorium intybus* L.; watermelon, *Citrullus lanatus* {Thunb.} Matsum. & Nakai; mustard, *Brassica juncea* {L.} Czern.; and soybean, *Glycine max* {L.} Merr.], and (iv) apply the model to recordings from these plants to demonstrate the value of the method.

## Materials and Methods

### Training Dataset

**Fig. 1.**
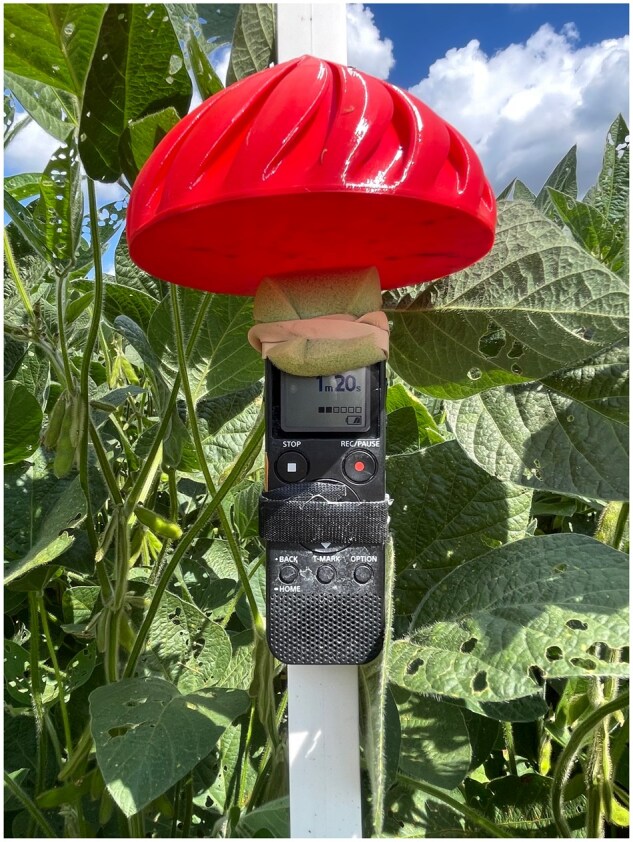
An example of a recorder deployed in soybean according to the bioacoustic methods developed by Forrester et al. (2024).

We produced the training audio for the machine learning model according to bioacoustics methods developed by Forrester et al. (2024). In brief, handheld Sony ICD-PX370 recorders were fastened to plastic electric fence posts, set at the height of the flowers of interest, and left to record for several days (Fig. 1). Recordings were saved as 48 kbps MP3 files with a 44.1 kHz sample rate. All training audio was produced in soybean fields during flowering in central Ohio. Audio events were manually annotated to serve as training data for the machine learning model. Audio events were identified using Audacity (v3.7.4, www.audacityteam.org) to listen to recordings while inspecting the spectrogram and comparing putative buzzes to reference audio. Audacity label tracks were used to produce annotations. In total, roughly 24,000 s of annotations were generated (Table 1).

**Table 1.** Training data volume by event type

Label	Description	Events	Seconds
**ambient_background**	Uneventful background noise	1,503	5,322
**mech_auto**	Cars, trucks, motorcycles	1,268	5,106
**mech_plane**	Jet planes, propeller planes	149	4,465
**ins_buzz**	Any insect flight buzz	1,570	2,508
**ins_trill**	Crickets and cicadas	336	1,915
**ambient_noise**	Loud ambient noises: scraping foliage, rustling leaves	816	1,414
**mech_hum**	Monotonous mechanical droning	68	1,283
**ambient_rain**	Rain and thunder	54	1,141
**human**	Human vocalization: talking, laughter	243	675
**frog**	Any calling frog	201	310
**mech_siren**	Emergency vehicle sirens	161	176
**mech_train**	Train horns	83	100
**bird_goose**	Canada goose calls	27	31

The “label” column gives the names of the labels used to annotate the training set. Each label has a corresponding output neuron for classification. Data volumes for each label are given by number of discrete events and by the total seconds of audio.

We labeled around 2,500 s of buzzing insect flight in the training data. Because it was not possible to precisely identify insects from the audio alone, we grouped buzzes into 3 broad categories according to the pitch relative to the buzz of honey bee (*Apis mellifera* L.) flight, about 230 Hz (Altshuler et al. 2005), for which we had the best reference audio. Eight percent of the training data for insect buzzes was from buzzes with a pitch higher than honey bees, 58% from medium-pitched buzzes (those that we found to be a good match to our reference audio), and 33% from buzzes with a lower pitch. While a bioacoustic model capable of automatically identifying flying insects is highly desirable, our current dataset is not sufficient to train a model to distinguish between buzz pitches with useful accuracy. Thus, we have trained our current model to detect insect buzzing in general by conflating all buzz pitches into the single label “ins_buzz.”

### Model Training

We employed transfer learning to train a convolutional neural network using a relatively small volume of data. In brief, this technique entails acquiring a pretrained, general-purpose model and retraining a small part for a specific task rather than attempting to train an entire model from scratch. The general structure for model training followed the “Transfer learning with YAMNet” tutorial by the Google TensorFlow team (TensorFlow Authors 2022). We used the pretrained audio classifier YAMNet (Plakal and Ellis 2025), which was trained on the AudioSet corpus (Gemmeke et al. 2017) to classify audio into 521 events. However, these events are largely irrelevant to pollinator study systems. We created our own model in order to fine-tune detection for field audio. Our model functionally replaces the final layer of YAMNet, which performs classification. We use the values from the second-to-last layer of YAMNet (the embedding layer, see TensorFlow Authors 2022) as the input and connect these values to 8 output neurons corresponding to our events of interest (see Table 1). We then trained our model by applying YAMNet to our training dataset, extracting values from the second-to-last layer, and training our model on the extracted values.

### buzzdetect

Accompanying the model, we developed a Python-based tool to facilitate the analysis of large volumes of audio data. We call the tool “buzzdetect” (stylized in the lower case). To accelerate analysis, buzzdetect employs parallel processing and optional processing on GPU. Audio files to be analyzed should not require any preprocessing: we use the packages soundfile (Bechtold 2025) to read a wide variety of file formats and librosa (McFee et al. 2023) to resample audio in-memory to the appropriate sample rate. On modest hardware (Intel Core i7-2600 8-core CPU, NVIDIA GeForce GTX 1650 GPU, 7200 RPM Western Digital HDD), we see an analysis rate of 3,600×, analyzing an hour of audio every second. On an M1 Macbook Air, we see an analysis rate of roughly 1,000×.

YAMNet segments input audio into discrete 0.96-s “frames” and performs classification on each frame. For each input audio file, buzzdetect outputs model results to a CSV file as follows: each row corresponds to a single audio frame, a “start” column records the timestamp of the start of the frame in seconds, and a column for each output neuron records the activation value of the neuron for that audio frame.

An output neuron’s activation is a measure of the model’s confidence that the neuron’s corresponding event is present in the input data. Thus, where the “ins_buzz” neuron activation is high, we expect to hear the buzz of insect flight. The researcher then sets a threshold above which to call detections. The choice of a threshold involves a tradeoff between sensitivity and false positive rate. We use sensitivity to mean the probability of a detection given the occurrence of insect flight buzzing in the frame (also termed “recall”). The false positive rate is the proportion of negative examples for which a positive prediction is generated (a nonbuzz called as a buzz). While the false positive rate gives a sense of the model’s error, it does not itself provide information on the signal-to-noise ratio. For this, we calculated the model’s precision: the probability that a buzz is present in the frame given a detection. A higher threshold is more conservative; it will produce a lower false positive rate (nonbuzzes are unlikely to be called as buzzes), yielding a higher precision (the called detections are more likely to be true) at the cost of sensitivity (true buzzes are less likely to be called). buzzdetect can output results as the raw activation values of the neurons, or users can specify a precision level. If the user specifies precision, the threshold is automatically calculated from the model’s testing data and results are returned as binary detections for each frame. We propose calling detections at a threshold corresponding to 95% precision. We have found that lower precision values can produce implausible trends due to false positives, such as spikes of buzz activity during the night. The exact threshold for 95% precision will vary between models, but we include the value with each model we release.

### Model Testing

To evaluate the model’s performance in a realistic, long-term context, we selected a small subset of audio files from our previous unpublished work in 4 crops (pumpkin, watermelon, mustard, and soybean) and 1 wildflower (chicory) located in central Ohio. None of the audio used for evaluation was part of the model’s training set and all of the test recordings were conducted at different sites than the training recordings. This provides a truly out-of-sample test set with real-world audio event distributions and distinct environmental noise profiles.

Each of these studies followed the bioacoustic methods developed by Forrester et al. (2024) and described in the “Training dataset” section above. From each experiment, we selected audio from 8 randomly selected recorders on a single day near the middle of the flowering period: 8 August 2024 for pumpkin, 4 July 2025 for chicory, 27 July 2024 for watermelon, 9 September 2024 for mustard, and 10 August 2023 for soybean. This produced eight 24-h recordings from each plant for a total of 40 recordings. We then selected 1 recording per crop and manually annotated the audio events contained in the first 5 min of every hour of the recording from 6 AM to 8 PM. This produced a total of 75 manually annotated time periods for comparison to the model results. Our annotations for the testing data followed the same labeling scheme as for the training data, with the addition of “quiet” and “loud” tags to evaluate how the model is affected by the volume of a buzz. We considered a buzz “quiet” if it was barely visible on the spectrogram and not easily heard without increasing the audio volume; the “loud” tag was used when a buzz stood out strongly on the spectrogram and was much louder than any other noise occurring at that point (Supplementary Fig. S1). Sometimes a buzz contained quiet and loud portions; we labeled these sections independently and remerged them in code for per-buzz analyses. At some points the insect activity was so high that it produced a nondistinct and constant background hum of activity. This occurred in mustard from 10 AM to 3 PM and in soybean from 12 PM to 2 PM. The spectrogram in these audio files was enriched in bands that matched the medium buzz pitch. In these cases, we tagged the whole duration as “background.” In mustard, the audio at 9:00 AM appeared to contain a background hum, but the sound was faint and often drowned out by overlapping sound. It was unclear whether or not this should qualify as a positive example of insect activity. We opted not to annotate the background hum for general analysis, but we examined how this decision affected the false positive rate in mustard.

We matched every frame of the buzzdetect results to their corresponding labels to assess model performance. A frame was considered positive for insect buzzing if 10% or more (0.096 s) of its duration overlapped with a buzz annotation or if the entirety of the buzz was contained within the frame. We calculated sensitivity, false positive rate, and precision across a wide range of threshold values. We investigated the response of these metrics to the deployments in different plants, to buzz pitch, and to buzz volume. We also examined how the ins_buzz neuron activation was affected when a buzz was overlapping with non-buzz sounds such as passing planes and cricket calls. While the sensitivity value is defined in terms of frames, we also calculated the per-buzz sensitivity by finding the probability that a buzz was detected in at least one frame for its duration and we investigated the effect of buzz duration on per-buzz sensitivity.

### Application

To simulate a research application of buzzdetect, we applied the model to the full 24-h recordings. Here, we intend to briefly demonstrate a possible application of buzzdetect and analyses of the results. With a single site and single day of observation for each plant, this experimental design is not capable of identifying fundamental ecological differences between these environments.

We used a threshold corresponding to 95% precision, as determined by our model testing results, and calculated the detection rate across time by binning results into 10-min bins and dividing the number of positive frames by the total number of frames in the bin. Using the R (v4.5.1, R Core Team 2025) package ggplot2 (v3.5.2, Wickham 2016), we plotted the trend of each recorder across the day to show the qualitative pattern of diel foraging in each crop.

We tested for differences in the average foraging intensity in each plant by calculating the average detection rate across the course of the day and regressing this against plant type in a beta regression using the R package betareg (v. 3.2.3, Cribari-Neto and Zeileis 2010). To correct for differences in the model’s sensitivity between different plants, we scaled each recorder’s detection rate according to the sensitivity achieved in that plant as determined during model testing. Differences were tested in a pairwise post hoc test using the pairs.emmGrid function of the package emmeans (v1.11.2, Lenth 2024) and applying the Tukey–Kramer method (aka “Tukey’s Honestly Significant Difference”) for multiple testing correction.

We tested for differences in the timing of foraging in each plant by determining the 10-min bin with peak foraging for each recorder. We expressed the time of day as a decimal with 0 and 1 representing midnight and 0.50 representing noon. Time of peak foraging was regressed against plant type in a linear model using the base R function lm and differences between plants were again tested with a pairwise post hoc test using the pairs.emmGrid function of emmeans with Tukey–Kramer multiple testing correction.

## Results

### Model Testing

**Fig. 2.**
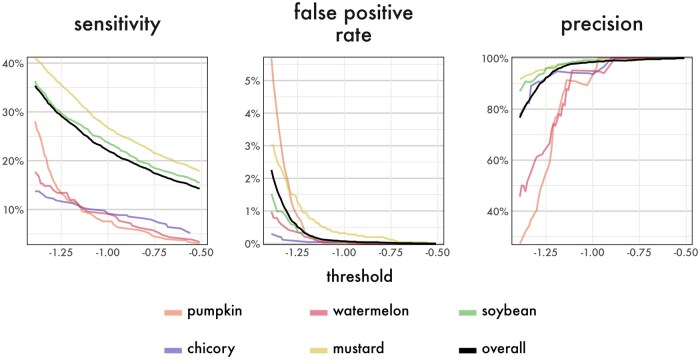
Trends for sensitivity, false positive rate, and precision across a range of reasonable threshold values. Crops are shown by color. The “overall” metrics are calculated by combining all data and are shown in black.

The tradeoff between sensitivity, false positive rate, and precision across thresholds can be seen in Fig. 2. Averaged across all plants and buzz pitches, at a threshold of −1.3, the model achieves 90% precision and detects buzzes with a 30% per-frame sensitivity; at a threshold of −1.2, the model achieves 95% precision and 28% sensitivity; at a threshold of −0.9, the model achieves 99% precision with 20% sensitivity. For all following results, we use the 95% precise threshold of −1.2, as this is our recommended level of precision for analysis. Sensitivity and precision varied between plants (Table 2). Sensitivity was highest in mustard (34%) and soybean (28%), but was near 10% in the other plants. Precision was near 95% for all plants except pumpkin and watermelon (both ∼75%). The false positive rate was below 0.35% for all plants except mustard (0.95%). However, most of these false positives were produced during the period where a background hum was faintly present (see the Model Testing section in Materials and Methods). If we considered all frames from this recording positive, the false positive rate for mustard was 0.59%. Across all recordings, the 95% precise threshold produced 64 false positives out of 20,833 nonbuzz frames for a total false positive rate of 0.3%. Mechanical sounds produced the most false positives, with 15 from planes (out of 2,115 frames for a 0.7% false positive rate) and 10 from ground vehicles (of 1,746 frames, 0.6%). Cricket calls produced 18 false positives (of 5,538 frames, 0.3%). Ambient background noise produced 20 positive frames (of 10,834 frames, 0.2%). See Supplementary Fig. S2 for examples of false positives.

**Table 2.** Performance metrics for each plant type and for all results combined

Category	Precision (%)	Sensitivity (%)	False positive rate (%)
**Pumpkin**	75	12	0.31
**Chicory**	94	11	0.05
**Watermelon**	74	12	0.20
**Mustard**	97	34	0.94
**Soybean**	96	28	0.32
**Overall**	95	28	0.31

Note that the false positive rate in mustard includes positives for the 9 AM background hum (see “Model Testing” in “Methods”). If this background hum is considered a true positive, the false positive rate drops to 0.59%.

Buzz characteristics strongly impacted sensitivity (Table 3). Sensitivity was similar in response to low (26%) and medium (29%) pitched buzzes, but the model was insensitive (2%) to high-pitched buzzes. Most buzzes observed were in the medium category. Sensitivity increased with buzz volume from 5% in response to quiet buzzes to 15% in response to normal buzzes to 56% in response to loud buzzes. In terms of per-buzz sensitivity, longer durations produced a greater chance detection. Most buzzes (53%) were around 1 s long, and the per-buzz sensitivity to this duration was 33%. Very short buzzes, such as by an insect passing the microphone but not foraging nearby, produced less sensitivity at 12%; these represented 15% of annotated buzzes. The longest buzz observed was over 12 s in duration.

**Table 3.** Model sensitivity to various types of buzzes and the proportion of buzzes associated with each type

Category	Type	Sensitivity (%)	Proportion of buzzes (%)
**Buzz pitch**	Low	26	5
	Medium	29	86
	High	2	9
**Volume**	Background	22	(dropped)
	Quiet	5	26
	Normal	15	69
	Loud	56	5
**Duration (sensitivity per buzz)**	<0.5 s	12	15
	0.5–1.5 s	33	53
	1.5–3 s	60	21
	>6 s	69	11

The pitch and volume categories use per-frame sensitivity; the duration category uses per-buzz sensitivity.

**Fig. 3.**
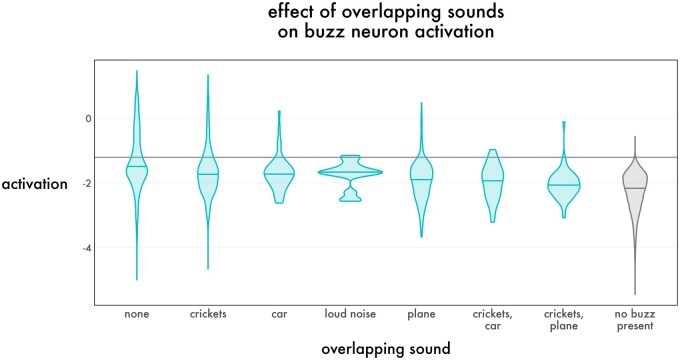
Distribution of ins_buzz neuron activations for buzzes in isolation (labeled as “none”), for cases where another sound overlapped the buzz (labeled with the type of sound), and for nonbuzzes. Violin plots are used due to the strong nonnormality of the distributions; center lines represent median activation. The horizontal blue line represents the 95% precise threshold of −1.2; activations above this line would be called as buzzes.

The presence of overlapping sound meaningfully lowered detection sensitivity (Fig. 3). Isolated buzzes with no overlapping noise produced a sensitivity of 36% (number of frames = 2,246 for 55% of all buzz frames). The presence of cricket calls decreased sensitivity to 21% (*n* = 1,393, 34% of buzz frames). Louder events such as passing cars (*n* = 49, 1% of buzz frames) and planes (*n* = 293, 7% of buzz frames) decreased sensitivity to 10% and 6%, respectively. Multiple overlapping sounds appeared to drive sensitivity lower: when both cricket calls and passing vehicles were present, sensitivity was still lower at 4% (*n* = 74, 2% of buzz frames). On the other hand, overlapping buzzes greatly increased the probability of a positive frame: when a frame contained more than one source of buzzing and no nonbuzz sound, sensitivity increased to 70% (*n* = 558, 14% of buzz frames). See Supplementary Fig. S3 for examples of false negatives.

### Application

**Fig. 4.**
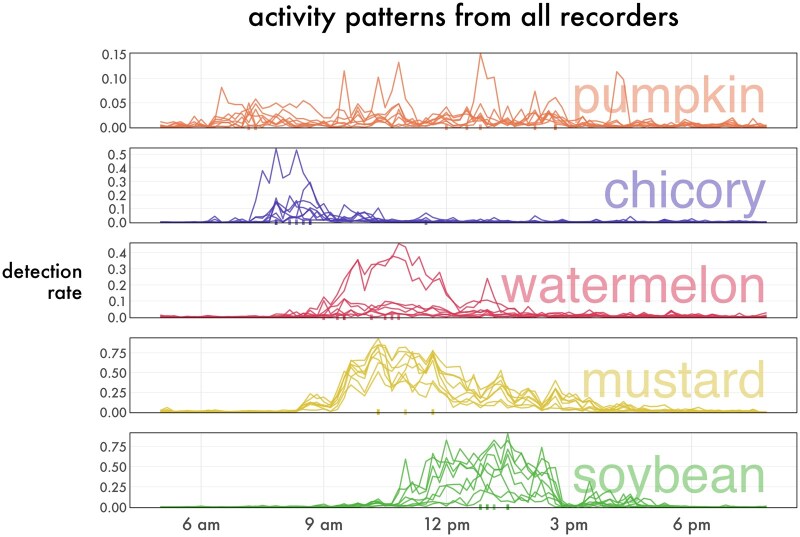
Activity curves for each of the 8 recorders for each crop. Detection rate is calculated in 10-min bins. Hash marks on the *x* axis represent peak foraging time of one of the recorders.

Mustard showed significantly higher foraging activity than all other crops (all *z *> 3.7, all *P *< 0.01). Soybean showed higher foraging activity than pumpkin (*z *= 4.5, *P *< 0.01), chicory (*z *= 4.42, *P *< 0.01), and watermelon (*z *= 4.08, *P *< 0.01). Pumpkin, chicory, and watermelon did not significantly differ in their total detected activity. Average foraging activity varied considerably within the same crop (Fig. 4). Two recorders in watermelon showed much greater activity than the others, with 4,091 detections from recorder 1_147 and 3,943 detections from recorder 1_73 over the 24-h period. The other recorders in watermelon averaged around 1,200 detections in the same period. This difference was genuine, not a model artifact, as a review of the audio around peak foraging confirmed the presence of many buzzes. Even in mustard where the recorders showed the most consistent trend in foraging activity, detection rates spanned a range of roughly 0.50 during the period of peak activity.

These results also reveal differences in the timing of foraging between these plants. The timing of peak activity was later in soybean than in chicory by about 4.5 h (*t*_35_ = 5.9, *P *< 0.01), later than watermelon by about 3 h (*t*_35_ = 4.2, *P *< 0.01), and later than mustard by about 2.5 h (*t*_35_ = 3.2, *P *< 0.03). Peak foraging in chicory was earlier than foraging in pumpkin by about 3 h (*t*_35_ = −4.2, *P *< 0.01), but we note that the peak foraging time model shows nonnormality in the residuals per inspection of the *Q*–*Q* plot (Supplementary Fig. S4). The greatest outliers are from the late-peaking pumpkin recorders; removal of pumpkin from the dataset does not alter the significantly later timing of soybean activity and supports earlier foraging in chicory than in mustard (*t*_28_ = −5.4, *P *< 0.01) and watermelon (*t*_28_ = 3.3, *P * =  0.01). While most recorders in pumpkin showed early foraging peaks, recorder 1_37 saw a large spike in buzz detections around 4:10 PM, when pumpkin flowers are expected to be closed. Examination of the audio during the 10 min of peak detection found that all 61 of the frames were positive for loud, persistent high-pitched buzzing.

## Discussion

### Model Testing

The similar false positive rates across deployments suggests that the model is not susceptible to false positives arising from differing acoustic environments. All false positive rates are fairly low, but there may still be sounds that produce systematic false positives. We have previously observed that extraordinarily loud cricket calls severely distort the MP3 audio and can produce false positives (see Supplementary Fig. S2 for an example of distorted audio). We have noted that the sound of a quadcopter drone sounds remarkably similar to a bee buzz in recordings, but we have not tested the false positive rate from this sound. While we attempted to correct for the differing sensitivity between plants, this issue hinders direct comparison of activity levels. Differing levels of background noise could contribute to this effect, causing all buzzes to be slightly obscured in louder environments. There may also be an effect from the pollinator community at the different sites. Sensitivity is strongly affected by buzz pitch, so different assemblages of pollinators will produce different average sensitivities. However, medium-pitched buzzes were predominant in all sites and we believe that most of these were honey bees. Whatever the source of differential sensitivity, it must be accounted for in studies that seek to compare levels of insect activity between plant types, environments, or geographic regions.

The lack of sensitivity to high-pitched buzzes is likely due in part to the different characteristics of these buzzes—they nearly always sounded erratic and quavering, not like the steady buzz of bumble and honey bees. From observations in the field, we believe these buzzes may be from flies. High-pitched buzzes were also more likely to be quiet and short, 2 factors that lead to decreased sensitivity. However, the spike in the detection rate from long, loud, high-pitched buzzes in pumpkin demonstrates that the model is not categorically insensitive to this pitch. This strong difference in sensitivity is unfortunate for research targeting small-bodied insects. It also presents a challenge to future models which seek to classify insects by their flight buzz, as such models may be biased toward certain species. However, a similar problem is inherent to all bioacoustic methods: louder and more active organisms are by their nature more detectable. The buzz of a bumble bee propagates farther than the buzz of a mosquito. The bird that sings incessantly will produce more detections than the one that remains silent. Given this fundamental constraint, even if the underlying model were flawless, careful interpretation is required to relate the numerical results to the biological phenomenon of interest.

The level of foraging activity itself can affect model sensitivity. Sensitivity tripled when multiple buzzes were present in a frame, and buzzes are more likely to overlap when activity is high. This property is not necessarily a shortcoming, as it produces higher detection rates when numerous insects are present even if their buzzes overlap in time. When nonbuzz sounds overlapped with buzzes, performance was significantly degraded. In fact, our measurement of this effect is an underestimate, since passing planes and cars could be so loud as to obscure buzzes even from the human labeler. Such acoustic pollution represents a challenge to bioacoustic studies, but is not inherently different from other nuisance variables and blocking effects.

### Application

While exact times vary by study location, the diel patterns of foraging captured by buzzdetect are corroborated by existing literature. Foraging in watermelon (Njoroge et al. 2010, Di Trani et al. 2022, Korichi et al. 2025), pumpkin (Ali et al. 2014, Phillips and Gardiner 2015), and other cucurbits has been shown to peak early in the morning and taper off by midday. Foraging in soybean sees a more gradual onset and offset later in the day (Chiari et al. 2005, Blettler et al. 2018, Jung et al. 2020, Souza et al. 2023, Forrester et al. 2024). In chicory, flowers open early and remain open for only a short period; Barcaccia et al. (2016) state, “anthesis is complete before 10 AM. The flowers usually do not stay open after the morning.” The trend in mustard is less distinct, but a midday bloom with high visitation is also supported by existing literature (Mandal et al. 2018, Gautam et al. 2022, Adlin Prajula et al. 2023, Priyadarshini et al. 2025). The late detections in pumpkin are intriguing given that the flowers were undoubtedly closed by 4 PM. We are not certain as to the identity of the insect producing the buzz, but the steady high pitch suggests a small insect capable of hovering in place, perhaps a species of syrphid fly.

The differences in total activity are likely due in part to floral attractiveness, and the results correlate well to our observations in the field. However, they could also be driven by seasonal effects, locational effects, floral density, or even different foraging behavior at the flower. With this small sample, we seek to demonstrate the potential of buzzdetect to identify foraging patterns rather than to draw firm conclusions as to their drivers in these particular plants.

**Fig. 5.**
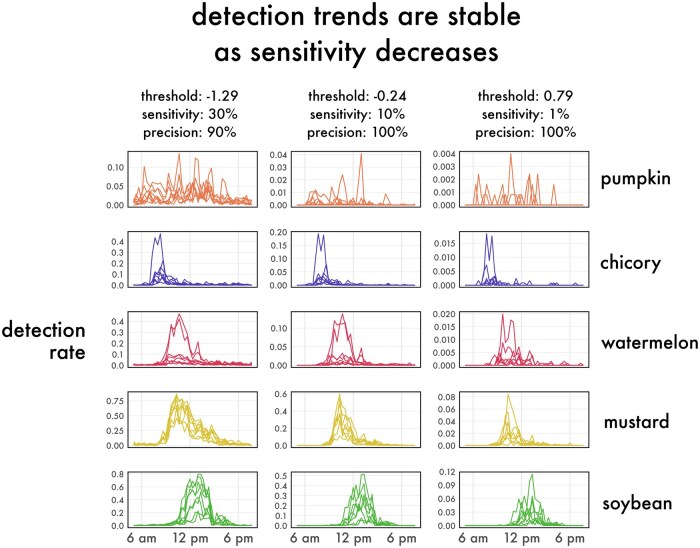
Trends in activity curves are similar even at extremely conservative threshold values. In plants with a clear peak of activity, the peak time is similar across these 3 thresholds.

We found that the general trend of activity over the course of the day looks similar even at remarkably low sensitivities (Fig. 5). When analyzing every second of the day, even a low sensitivity is sufficient to capture an informative sample. With the 95% precise threshold, the number of detections our model produced in a day on average was about 3,600. Comparing this to the normal abundance of capture in pan traps and sweep nets, the realized sensitivity of this method may be comparable to other sampling methods. For example, Hudson et al. (2020) found that only about 20% of bees that approached a pan trap were successfully captured by the trap.

### Considerations for Use

While our model can detect insect buzzes, it is not able to identify the insect producing the buzz. Distinguishing between species by their flight buzzes is a more challenging task than discriminating buzzes from environmental noise, and preliminary testing showed that our dataset was not sufficient to train a model to make such a distinction. Prior work has produced models capable of classifying insects given a positive example of a buzz (eg Gradišek et al. 2017, Ribeiro et al. 2021, Ferreira et al. 2023), and buzzdetect could serve as a first-pass to extract buzzes for further identification by these more specific models.

Diminished sensitivity from overlapping sound is problematic, but not uniquely so. This represents a new instance of an old problem: nuisance variables and confounders that can be addressed by increased sampling, statistical deconfounding, and careful study design. Our preliminary data show that signal can be extracted even from low-activity sites by increasing sample size. High replication is made easier by the automated nature of buzzdetect; it often takes longer to drive to the field site than it does to set up the recorders. Experimenters should block treatments across known gradients of noise pollution. For example, if one transect of research plots borders an active road, an even number of treatment and control sites should be assigned along the road.

At 48 kbps MP3, our training, testing, and application audio is highly compressed. This affords much longer recordings at the cost of audio fidelity. To ensure that buzzdetect is performant with other audio codecs and microphones, we conducted a brief test with an AudioMoth recorder (v1.2.0, Open Acoustic Devices) recording 44.1 kHz WAV audio. We placed the microphone at various distances from the entrance of a honey bee hive to produce different detection rates, then compressed the audio into MP3 and Vorbis OGG at various bitrates. Detection trends were nearly identical for all bitrates except for MP3 at 32 kbps, which is the lowest bitrate supported by the codec (Supplementary Fig. S5). Although our model was trained on 48 kbps MP3 data, performance is similar and may be superior with higher bitrates or with uncompressed audio. We suspect, but have not rigorously tested, that higher bitrates will ameliorate the effects of overlapping sound on model sensitivity; it can often be clearly seen in the spectrogram that loud events destroy information in other frequency bands, a result of lossy compression.

Differential sensitivity across environments and plant types presents an issue for research seeking to compare levels of foraging between these factors. The difference may be driven by an underlying difference in pollinator assemblage, with some environments containing a greater proportion of buzz types to which our model is less sensitive. Because our training and testing data are all from Ohio environments, it is possible that performance will vary across geographic regions due to differences in the insect species present. For example, our model would register fewer detections in a site with much activity from high-pitched flies compared to one with half as many honey bees. Our training data contain a variety of buzz pitches presumably from a variety of insect species, but it is possible that the model is fit to our local species in particular rather than insect buzzing in general. Imperfect detection pervades all sampling methods (Chen et al. 2013, Kellner and Swihart 2014), but it is not without redress (Royle et al. 2005, Guillera-Arroita 2017). It is beyond the scope of this article to fully address imperfect detection arising from bioacoustics (see Ducrettet et al. 2025 for detailed discussion), but we offer some practical advice for handling this problem below.

Validation of buzzdetect results from a new environment can follow a similar approach to the one described here. Namely: select a subset of recorders for human annotation, extract an even subsample of audio data from across the course of the day (or days), manually annotate the subsample, compare the annotations to the model results, and adjust detection rates accordingly. Our analyses are fully reproducible from the scripts and data deposited to Zenodo. The depth of sampling should be considered carefully. In this paper, we selected a single recorder from each site, as all microphones for a given crop were deployed in the same site on the same day. However, an experiment with higher heterogeneity of sites will require a larger sample for validation, as will deployments in areas with lower insect activity. Sampling intensity is a function of the number of recorders selected, the number of subsamples taken across the day, and the duration of each subsample. We chose 1-h sampling intervals for the sake of convenience and 5-min samples to provide sufficient data. However, it would provide a better estimate of variance to sample more recorders with equivalently shorter samples. To estimate the confidence of the model’s sensitivity in an environment, a 95% confidence interval can be constructed from the comparison of model results and the annotated data, e.g. with the binom.test() function in R. The amount of audio required for a confident estimation depends on the frequency of buzzes in the sampled data, but using binom.test() and assuming the underlying sensitivity is 28%, 300 buzzes will estimate the sensitivity within ±5%. A more sophisticated approach would be to construct a beta regression with sensitivity as the outcome, site as the predictor, and each recorder as an observation and then use the confidence interval of the site parameter.

With this said, we note that estimation of the model sensitivity is only required to compare absolute detection rates, and only when the factor of interest cannot be randomized or blocked across sites. If treatments have been assigned to sites in a random or blocked fashion, differential sensitivity is reduced from a confounder to a nuisance variable, adding noise but not biasing the effect estimates. While we always encourage checking model results, there are a number of interesting research questions for which the absolute foraging rate is not of interest, but instead the relative trend in activity across time (demonstrated in the model of peak foraging times).

It is also difficult to translate bioacoustic detections to pollination services. Our model detects the buzz of insect flight—while changes in flight activity should correlate well to changes in pollination activity for a given plant, the correlation is not the same between different plants. For example, a honey bee foraging on a mustard flower must make numerous short flights to obtain a small amount of resources. Comparatively, a bee visiting a squash flower may land, walk about, and consume nectar for a long duration without making a single buzz. While the duration of bee presence is identical in both scenarios, the mustard bee will produce many more detections. It may be possible to create an index for flowers that correlates the number of buzzes to the rate of visitation, and this would be an interesting direction for future work, but this information is not currently available. Finally, the differences between flowers can demand different methodologies, which is likely to bias results. For example, it is easy to deploy a microphone in the midst of a cluster of soybean flowers, but the large blossoms of cucurbits require the recorder to be placed at a greater distance from the nectaries and pollen. Thus, the buzzes from soybean visitation may be louder on average than those from the cucurbits, increasing detections.

To address these shortcomings, we recommend that bioacoustic monitoring be supplemented with traditional sampling. A light sampling effort with visual observation or sweep netting could provide a ground-truth to compare with acoustic detections while still capitalizing on the labor savings of automated monitoring. These methods complement one another: bioacoustics provides intensive, time-rich, automatic monitoring, while traditional sampling provides rich taxonomic information and direct observation of pollinator activity. Rather than replacing existing methods of pollinator monitoring and insect sampling, bioacoustics adds another tool to the kit of the ecologist.

Like all sampling methods, our passive acoustic monitoring method is not without tradeoffs. In some applications, these tradeoffs may not meaningfully impact the results: deployment in patches where one species is the predominant forager, where pollinator assemblage is constant, or in order to examine the timing, but not absolute intensity, of insect activity. In other applications, we suggest a multifaceted approach. Passive acoustic monitoring is a rapidly evolving field and we believe buzzdetect is a promising first step in enabling automated, long-context, and large-scale monitoring of pollinators.

## Supplementary Material

ieaf104_Supplementary_Data

## Data Availability

buzzdetect is available at github.com/OSU-Bee-Lab/buzzdetect. All code and data used in this manuscript are available in the Zenodo repository at https://doi.org/10.5281/zenodo.15644083. We are developing a companion R package, buzzr, to enable efficient analysis of buzzdetect results; it can be found at https://github.com/OSU-Bee-Lab/buzzr.
