# Supplementary material for: buzzdetect: an open-source deep learning tool for automated bioacoustic pollinator monitoring
Source: J Insect Sci. 2025 Dec 10;25(6):ieaf104. doi: 10.1093/jisesa/ieaf104 (PMC12690740; doi:10.1093/jisesa/ieaf104)
Supplement: ieaf104_Supplementary_Data [file ieaf104_supplementary_data.pdf]

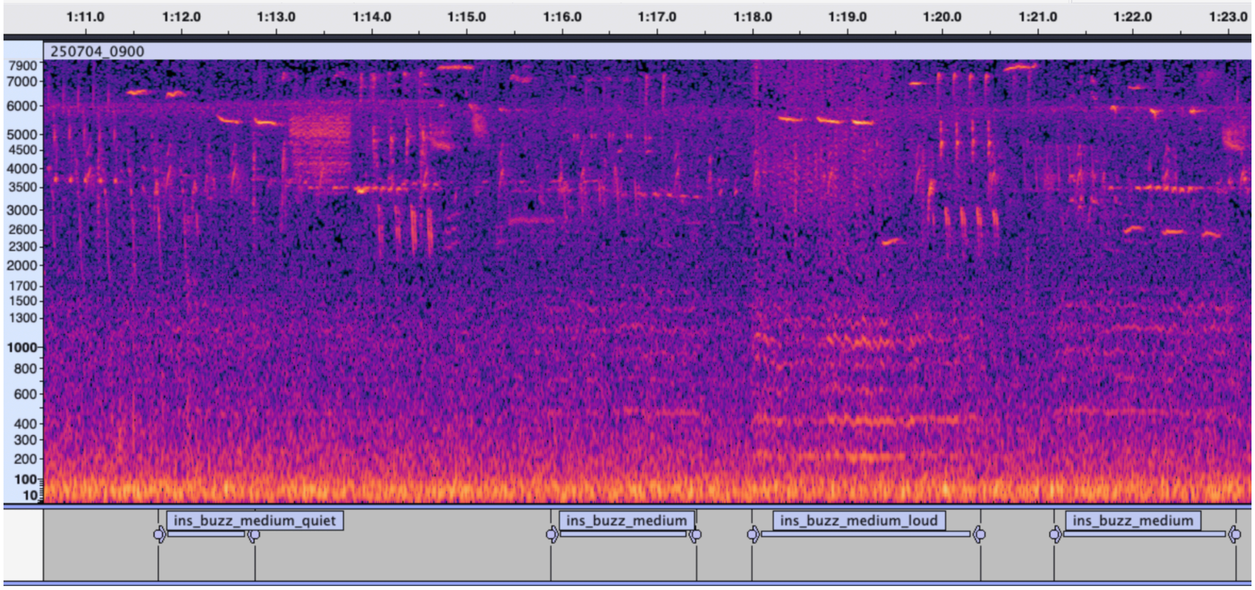

Supplementary Figure S1. Comparison of quiet, medium, and loud buzzes in the spectrogram view from Audacity. These results are for recorder 1_37 in chicory, file 250704_0900.mp3. Y axis is frequency in Hz, X axis is time in seconds. The quiet buzz is audible when the track gain is increased, but it only registers faintly compared to the background noise on the spectrogram. The loud buzz is clearly distinguished from the background noise.

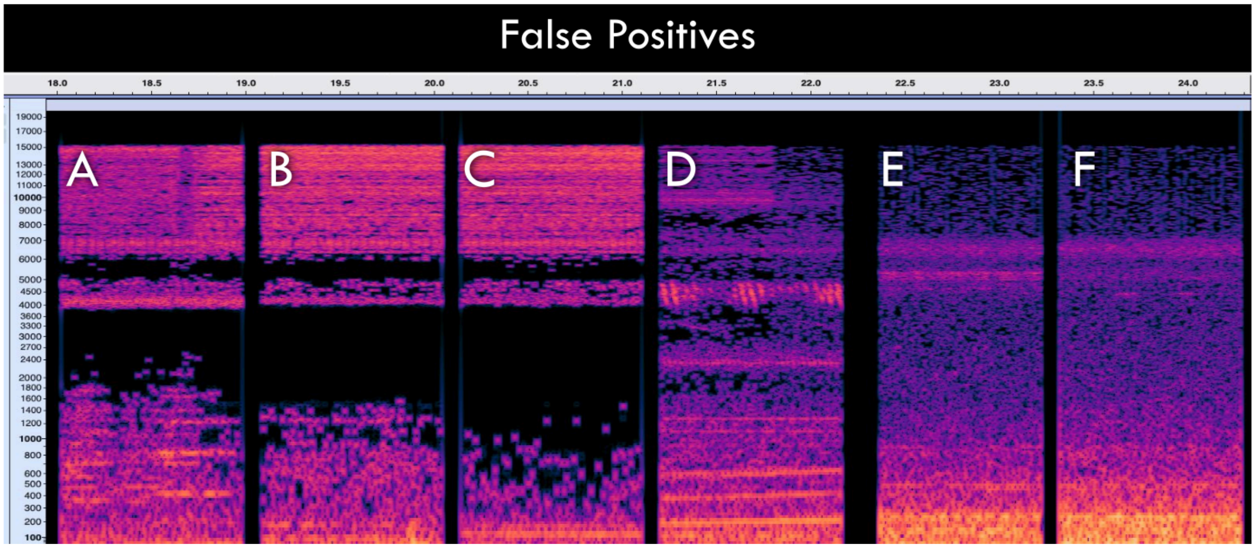

Supplementary Figure S2. Examples of false positive frames (erroneous detections). Most of the spectrograms superficially resemble insect buzzes. Frame A contains a car honk (~400 Hz, with harmonics) and cricket calls. Frames A–C contain cricket calls and the effects of MP3 compression can be seen where the high-pitched amplitude is destroying information in the lower bands). Frame D contains the sound of an engine revving. Frames E and F are ambient noise. This is a demonstrative sample and is not representative of the distribution of all false positives.

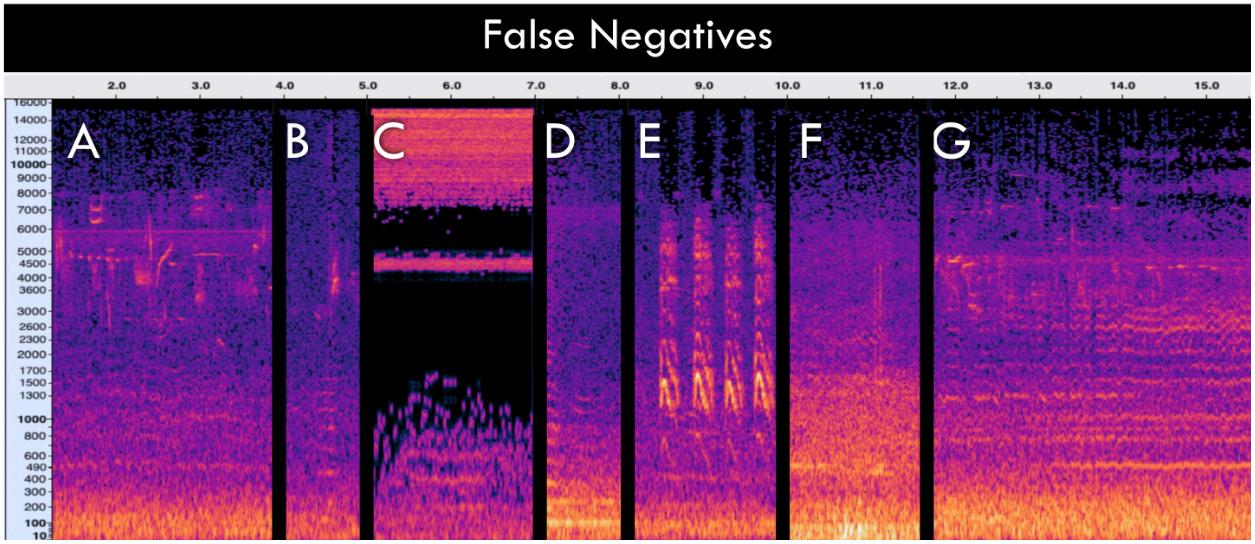

Supplementary Figure S3. Examples of false negatives (missed buzzes). Adjacent false negatives have been merged together; e.g., the portion of audio labelled A represents three consecutive frames with missed detections while B is a single missed frame. Section A shows a buzz that may have been missed because it was quiet. Section B shows a “fly-by” buzz, which is very short in duration. Section C shows a buzz overlapped by a cricket call, where the signal from the buzz is partially destroyed by MP3 compression. Only a small portion of Section D contains a buzz. Sections E and F are overlapped by environmental and mechanical noise respectively. The cause of a missed detection was not always apparent. Section G shows a clear and sustained buzz, yet none of the 4 frames yielded detections. This is a demonstrative sample and is not representative of the distribution of all false negatives.

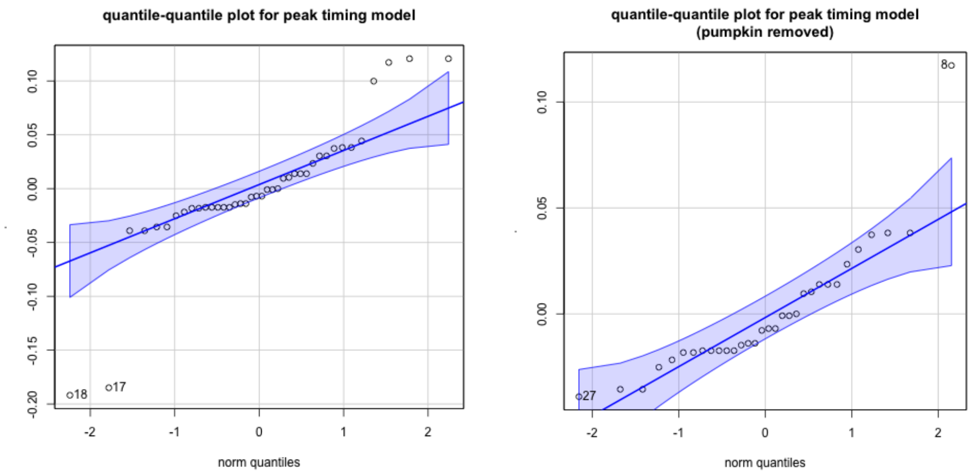

Supplementary Figure S4. Q-Q plots for the peak timing model showing goodness of fit. Outliers from pumpkin cause a poor fit to the data (left); removing them improves fit (right).

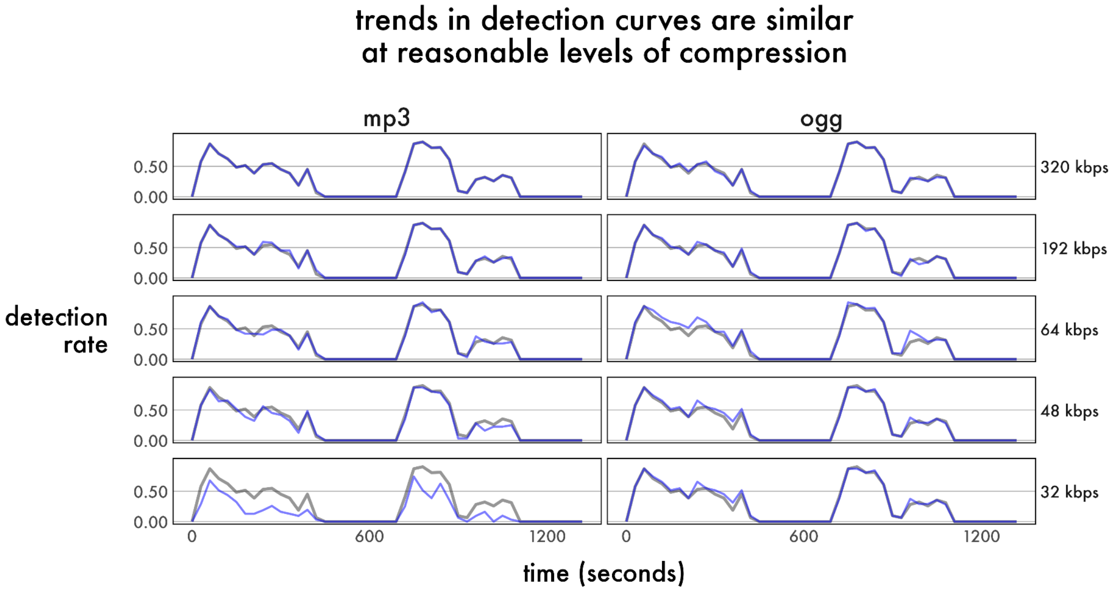

Supplementary Figure S5. The detection curves yielded by recording test audio in lossless WAV format at 44.1 kHz and compressing to different bitrates in two audio codecs, MP3 and Ogg Vorbis using ffmpeg. The gray detection curve in the background represents results for the uncompressed WAV audio. Trends in the curves remain nearly identical except for 32 kbps MP3, which is an extraordinarily high and lossy level of compression—192 kbps is common for MP3. The superior performance of OGG at 32 kbps likely reflects the higher efficiency of the codec; OGG is expected to have superior fidelity at a given bitrate compared to MP3. The test audio is available in the Zenodo repository for this article.

Supplementary Link S6. The raw data and scripts needed to reproduce the analyses in this study are available in the Zenodo repository at: https://doi.org/10.5281/zenodo.15644083
